# Core elements of character education essential for doctors suggested by medical students in Korea: a preliminary study

**DOI:** 10.3352/jeehp.2020.17.43

**Published:** 2020-12-21

**Authors:** Yera Hur, Keumho Lee

**Affiliations:** 1 Institute of Medical Education, College of Medicine, Hallym University, Chuncheon, Korea; 2 Center for Liberal Arts, Korea University of Technology & Education, Cheonan, Korea; Hallym University, Korea

**Keywords:** Character, Medical students, Professional practice gaps, Republic of Korea, Respect

## Abstract

This preliminary study aimed to determine how medical students perceive character education in Korea. A structured survey questionnaire was distributed to 10 medical students between September and December 2018, of whom 6 students replied. Students’ responses were classified into elements, which were also categorized. Twenty-nine core elements of characters in 8 categories were verified as essential for doctors and as needs for character education. The most frequently suggested categories were “care and respect,” “empathy and communication,” and “responsibility and calling.” Participants also stated that various forms of character education are necessary and that they were not satisfied with the teaching methods of the character education that they had received. These results verified the most essential character traits for doctors and identified problems related to current character education. The results of this study will be helpful for preparing the character education curriculum in medical schools.

## Background/rationale

To deal with regularly-occurring issues involving unethical and inappropriate behavior by doctors, it is necessary to examine which type of character education is necessary and how it differs from current education, as well as what current medical students think about character education.

## Objectives

This is a follow-up study of Hur and Lee [1] that aimed to examine how medical students perceive character education, with the specific goal of identifying and categorizing core character elements essential for doctors and perceived needs for character education.

## Ethics statement

This study was approved by the Institutional Review Board of Hallym University (HIRB-2018-049-1-C). Informed consent was obtained from the subjects.

## Study design

This was a preliminary survey-based observational study.

## Participants

To select appropriate medical students to respond to the survey, we arbitrarily selected a senior-year medical student who had recently won the grand prize of the Talent Award of Korea as a medical student. This student had some experience in medical education research, was a member of the student council, and was actively involved in club activities in and outside of school. She also had years of experience as a student lecturer in the medical humanities curriculum. From her social contacts, we reached out to 9 other medical students with experience in participating in the student council or as representatives in or outside their school. All participants had previously taken character education courses, although those courses may not have had the same format (content, instructional method, and evaluation method). The participants, who were in their 5th or 6th year of study, were selected from different medical schools in Korea and were asked to evaluate the content of the curriculum.

## Setting

This survey-based preliminary observational study explored the experiences of medical students in their senior academic years concerning character education. The 10 participants who agreed to participate in the study were sent structured interview questions between September and December 2018, to which only 6 students responded in written form. The lack of a response from 4 participants may have been due to their preparation for the medical licensing examination, as some participants were in their final academic years. The respondents included 1 female and 5 male students; 4 respondents were in their 5th year and 2 in their 6th year.

## Educational intervention

One should note that character education is acquired in various forms, including not only the formal curriculum, but also the informal curriculum and the hidden curriculum (e.g., teamwork activities, various club activities in and off-campus, and modeling professors’ behavior). This makes it difficult to clearly state the educational intervention of this study. Despite this limitation, Table 1 lists the subjects related to character education at the institutions with which the 6 participants were affiliated. The listed subjects are examples of formal programs that may have helped the students to experience character education.

## Measurement tool

The structured interview questionnaire consisted of 5 items: 3 open-ended items on character education used by Hur and Lee [1], and 2 additionally developed items (Supplement 1). This questionnaire was translated into English for other researchers (Supplement 2). Two additional questions (items 3 and 4) were developed to obtain more detail on medical students’ perspectives, focusing on the status of character education experienced by students as the consumers of medical education.

## Statistical methods

The interview results were summarized and categorized descitively (Dataset 1). No statiscal analysis was done.

## Outcome data

The results of the interviews with the medical students showed that all students considered character education to be necessary because character is the basic quality essential for doctors, as well as the model of a mature intellectual. Some students responded with the following opinions.

“Look at the social issues and cover-ups of all kinds of accidents. These problems occur even though doctors learned in theory from school that they shouldn’t do those kinds of things. Beyond just teaching knowledge and providing practical training, there is a need for maturity in terms of morality and personality.”
“It’s uncertain whether we can always maintain a consistent level of character as we study, even though we are adults. Medical education must provide not just knowledge, but also humanities and character education to guide us in the right direction with the competencies to graduate and maintain our basic qualities as doctors.”

According to the participants’ responses, they thought that the qualities of character most essential for doctors were a spirit of service and empathic ability. The main opinions were as follows.

“The most fundamental thing is a spirit of service. This is the spirit of making efforts to help and coexist with others, and not for personal gains.”
“There must be a holistic approach based on the empathic ability to determine the most applicable treatment for patients by understanding their conditions as well as possessing a comprehensive understanding of both the patients and their caregivers.”
“It is essential to think about patients and have a morally upright attitude without cheating.”

The respondents stated many opinions about the problems of character education in current medical education, most of which were related to theory-based education, apprenticeship learning, and unstandardized education. Some of the participants also mentioned inappropriate content of evaluations and theory-practice gaps.

“In a nutshell, there is no standardized education. Without establishing proper, standardized education, we won’t be able to break the vicious cycle.”
“Due to the issue of low medical reimbursement rates facing the medical system, students feel skeptical about the discrepancy between general perceptions of medical care and stated values of nobility, morality, and mission; and students hold onto their cynical attitudes.”

The students stated that there is a need for education focused on experiences or activities, education on communication and collaboration, and education focused on various cases to understand that the art of medicine is about the art of healing.

“Courses based on reading or debates rather than lectures, education focused on various activities such as community service off-campus.”
“I think there must be a humanities course that helps us understand patients’ thoughts and emotions as well as the situation they are in. Since doctors work with people in various professions at the hospital, we must learn about team-based collaboration.”

Students were generally satisfied with mentoring and education on communication skills, but not with education delivered through the old-fashioned methods of lecturing and reading.

“Character education that just provides a one-time lesson without any experience is meaningless.”
“The kind of character education I received was mostly boring lectures or quizzes on minor things after reading.”

## Main results

Thirty-two core elements were extracted from the question eliciting opinions on the core elements of character essential for doctors. This study incorporated 29 elements, excluding medical knowledge, expertise, and information power, which have little relevance to character, into the classification table developed by Hur and Lee [1]. The categories that included the most elements were “care and respect,” “responsibility and calling,” and “empathy and communication” (Table 2).

Participants also responded that various forms of character education are necessary and that they were not satisfied with the teaching methods used in character education. Furthermore, they stated that the problems related to current character education were theory-based education, apprenticeship learning, unstandardized education, inappropriate content of evaluations, and theory-practice gaps.

## Interpretation

The importance of character education in medical education has long been clear. However, studies among professors and students alike have indicated that there are negative perceptions regarding how character education is carried out [1]. It is necessary to consider not only the content, but also the teaching and evaluation methods of character education in medical education.

**Fig. 1.**
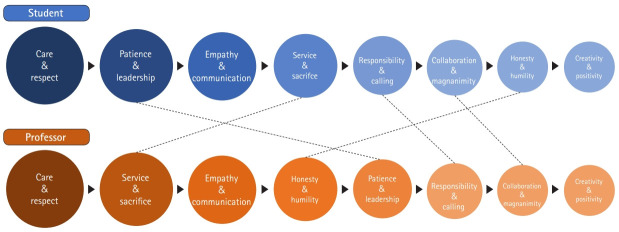
Comparing the importance of core elements of character education between students of the present study and professors in the previous study [1].

Fig. 1 shows the core elements essential for doctors chosen by the medical students. They chose “care and respect” as the most important core element, which is consistent with our previous study conducted among professors [1]. However, as pointed out by Beauchamp [2], in the evolving paradigm of post-modernity, professionals pursue a balanced life between their lifestyle and responsibility, accountability, and autonomy. Therefore, the “service and spirit of sacrifice” valued by professors in the framework of traditional professionalism is pushed to below 3rd place for the new millennial generation, which values work-life balance. The significance of this study is its examination of how medical students perceived character education using a structured questionnaire. The term “character,” which may be confused with “personality traits,” refers to the basic attitude, values, and mindset of a doctor [1]; as such, its meaning is closer to “virtue.” According to a study by Leffel et al. [3], virtuous students were recognized by their peers to be exemplary doctors, and virtues, rather than personality traits, predicted prosocial behavior. Hawking et al. [4] also stated that medical students face ethical and professional problems during clinical education. In the face of these problems, virtues such as wisdom, response, composition, or empathy are useful to medical students. Therefore, character education should be provided in medical school programs and should be considered in the design of educational experiences that intentionally emphasize the building of virtues. In other words, the virtues and proper behavior that a doctor should have can be fostered through character education.

## Limitation/generalizability

The generalizability of the results is limited, as this preliminary study examined only a few medical students. There are also limitations of the educational intervention of the study due to the differences in the 6 participants’ experiences of character education. The 4 schools provided common programs such as communication, leadership, mentoring, and professionalism, but some schools had character education-focused programs such as “character education practice,” and the number of subjects related to character education were different among the curricula. An in-depth follow-up study including interviews with a greater number of medical students should be done, and further research should also determine whether there are differences in perceptions or levels of character depending on demographic characteristics. It is difficult to generalize the present results to medical schools in Korea or abroad because of the preliminary nature of this study.

## Conclusion

The medical students who participated in this study suggested 29 elements of character education, which can be classified into 8 categories. Three categories were particularly prominent: care and respect, empathy and communication, and responsibility and calling. Society requires doctors who possess not only medical knowledge and skills, but also a high level of ethics, responsibility, and morality. It is necessary to develop and apply curricula, teaching methods, and evaluation methods for character education in medical schools to be practical and effective, instead of superficial or perfunctory.

## Figures and Tables

**Table 1.** List of subjects related to character education at participants’ medical schools

Medical school	Subjects related to character education
Medical school A^a)^	Medical humanities; Conversations with mentors; Doctors and professionalism
Medical school B^b)^	Leadership Development; Activities for human growth I, II; Leadership practice; Understanding and communicating with medical organizations; Patient, Doctor, Society
Medical school C^c)^	Flex mentoring; Motivation inducement; Foundation of medical humanities; Communication skills
Medical school D^d)^	Physician leadership; Character education practice; Medical ethics based on team-based learning

a) http://medicine.ajou.ac.kr/fr_university/sub_02_04.

b) https://medical.yonsei.ac.kr/we/index.php?mid=edu_course&category=1756&document_srl=1931.

c) https://med.konyang.ac.kr/med/sub02.01.do.

d) https://www.kmu-med.ac.kr:7454/content/04class/01_02.php?part=20.

**Table 2.** Categories and core elements of character education essential for doctors suggested by medical students in Korea

Category^a)^	Elements (no. of responses)
Care and respect (n=9)	Care (3), respect (2), companionship (1), understanding (1), understanding of patients (1), attention (1)
Patience and leadership (n=1)	Leadership (1)
Empathy and communication (n=8)	Communication (3), empathy (2), interaction (1), open mind (1), love for humanity(1)
Service and sacrifice (n=4)	Spirit of service (2), sharing (1), spirit of sacrifice (1)
Collaboration and magnanimity (n=6)	Collaboration (4), management (1), magnanimity (1)
Responsibility and calling (n=7)	Responsibility (3), effort (1), diligence (1), ethics (1), patience (1)
Honesty and humility (n=5)	Honesty (2), humility (1), fairness (1), order (1)
Creativity and positivity (n=2)	Flexibility (1), creativity (1)

a) The categories of the core elements were adopted from the classification by a previous study of Hur and Lee [1], “Identification and evaluation of the core elements of character education for medical students in Korea.”

## Footnotes

**Data availability** Data files are available from Harvard Dataverse: https://doi.org/10.7910/DVN/W4NOCU Dataset 1. Students’ responses to the structured questionnaire (in Korean).

## Supplementary materials

Supplementary files are available from Harvard Dataverse: https://doi.org/10.7910/DVN/W4NOCU

Supplement 1. Korean version of the opinion survey on character education administered to medical students.

Supplement 2. English translation of the opinion survey on character education administered to medical students.

Supplement 3. Audio recording of the abstract.

## References

[1] Hur Y, Lee K (2019). Identification and evaluation of the core elements of character education for medical students in Korea. J Educ Eval Health Prof.

[2] Beauchamp G (2004). The challenge of teaching professionalism. Ann Acad Med Singap.

[3] Leffel GM, Oakes Mueller RA, Ham SA, Karches KE, Curlin FA, Yoon JD (2018). Project on the good physician: further evidence for the validity of a moral intuitionist model of virtuous caring. Teach Learn Med.

[4] Hawking M, Kim J, Jih M, Hu C, Yoon JD (2020). “Can virtue be taught?”: a content analysis of medical students’ opinions of the professional and ethical challenges to their professional identity formation. BMC Med Educ.

