# Supplementary material for: Core elements of character education essential for doctors suggested by medical students in Korea: a preliminary study
Source: J Educ Eval Health Prof. 2020 Dec 21;17:43. doi: 10.3352/jeehp.2020.17.43 (PMC7897509; doi:10.3352/jeehp.2020.17.43)
Supplement: Supplementary file 2 — Supplement 1. Korean version of the opinion survey on character education administered to medical students. [file jeehp-17-43-suppl1.docx]

Suppl. 1. The questionnaires of the opinion survey on personality education of medical students

**의과대학생의 인성교육에 대한 의견조사**

| ◎ 연구의 목적  이 연구는 의학교육에서의 인성교육을 재정의하고 의사가 갖추어야 할 인성의 핵심 요소를 규명하여 의과대학생을 대상으로 하는 인성교육의 졸업역량과, 시기별 학습성과, 교육내용과 방법에 대한 기준을 제시하고 의과대학 교육과정 안에서 실제로 적용할 수 있는 인성교육 멘토링 모듈(가칭)을 개발하는 것을 목표로 합니다. 이번 조사는 의과대학생의 인성교육에 대한 의견을 조사하는 데 있습니다.  ◎ 용어의 정의  본 연구에서 의미하는 **인성**이란 좋은 의사가 되기 위해 갖추어야 할 윤리적/행동적 가치, 성품, 덕목, 인간상이라고 할 수 있습니다. 이는 좋은 의사에게 요구되는 지식, 기술, 태도를 모두 포함하는 포괄적인 개념의 의학전문직업성이나 의료인문학이라는 복합적인 개념과 겹치는 영역도 있겠으나, 동일하기보다는 구별된 차원으로 보는 것이 보다 바람직하겠습니다. 일부 선행연구에서는 인성을 성격(character)로 보는 견해도 있습니다. 의학교육계에서는 2014년 한국의 의사상 설정연구(보건복지부)를 통해 바람직한 의사의 역량을 ‘환자진료’, ‘소통과 협력’, ‘사회적 책무성’, ‘전문직업성’ 및 ‘교육과 연구’ 등 5가지 영역으로 규명하였고, 교육부의 2014년도 정책연구에서는 초, 중, 고등학교 인성교육의 핵심 요소를 자아정체감, 정직, 책임, 존중, 배려, 공감, 소통, 협동으로 발표한 바 있습니다.  ◎ 질문지 응답 방법  이 질문지의 가장 큰 목적은 개방형으로 형식의 제한 없이 응답자의 자유로운 의견을 많이 수집하는 것입니다. 이에 질문에 응답해 주실 때에는 가능한 **구체적인 내용**으로 풀어서 기술해 주시기 바랍니다.  예) 질문: ‘우리나라 의사에게 필요한 인성’  ⇒ 응답 예 : **타인을 존중하는 마음가짐, 타인을 위해 봉사하고자 하는 희생정신** |
| --- |

| **성 명** |  | **출생연도** |  |
| --- | --- | --- | --- |
| **소속대학** |  | **학년** |  |
| **개인 소개 및 의학교육 관련 활동/경력** |  | | |

1. [인성교육의 필요성] 의학교육에서 인성교육이 필요하다고 생각하십니까?

|  |
| --- |

2. [개념 정의] 의사에게 요구되는 인성적 소양 즉 **“인성”은** 무엇이라고 생각하십니까?

|  |
| --- |

3. [인성교육의 교육역량] 의사에게 요구되는 인성을 교육하기 위해서 어떠한 교육역량이 필요하다고 생각하십니까?

|  |
| --- |

4. [현황 파악] 현재 의학교육이 갖고 있는 인성교육에서 적절하게 시행되고 있는 것이 있다면 어떤 것이라고 생각하십니까? 그리고 어떤 점에서 적절한 인성교육이라고 판단하십니까?

|  |
| --- |

5. [문제점] 현재 의학교육이 갖고 있는 인성교육의 문제점이나 실패하고 있는 부분이 있다면 어떤 것이라고 생각하십니까?

|  |
| --- |

6. [방향성] 4차 산업시대의 의사가 갖추어야할 인성의 핵심요소를 **10가지 내외로 나열**해 주시고, 중요도 순으로 1번부터 10번까지 번호로 매겨주십시오.

| **번호** | **요소** | **구체적인 의미** | **중요도(1~10위)** |
| --- | --- | --- | --- |
| 응답 예 | 봉사 | 타인을 위해 봉사하고자 하는 희생정신 | 2 |
| 1 |  |  |  |
| 2 |  |  |  |
| 3 |  |  |  |
| 4 |  |  |  |
| 5 |  |  |  |
| 6 |  |  |  |
| 7 |  |  |  |
| 8 |  |  |  |
| 9 |  |  |  |
| 10 |  |  |  |

- 참여해 주셔서 감사합니다! -
