# Supplementary material for: Core elements of character education essential for doctors suggested by medical students in Korea: a preliminary study
Source: J Educ Eval Health Prof. 2020 Dec 21;17:43. doi: 10.3352/jeehp.2020.17.43 (PMC7897509; doi:10.3352/jeehp.2020.17.43)
Supplement: Supplementary file 3 — Supplement 2. English translation of the opinion survey on character education administered to medical students. [file jeehp-17-43-suppl2.docx]

Supplement 2. English translation of the opinion survey on character education administered to medical students

1. Do you think character education is necessary in medical education?
2. What are the qualities of character most essential for doctors - how would you define the “characteristics” need for a good doctor?
3. What kind of character education do you think is necessary for the doctor to have the characteristics required?
4. From your personal experience of character education in medical school, what are the programs that you are satisfied with, and why?
5. From your personal experience of character education in medical school, what do you think is the problem or failure of character education that medical education currently has if there is any?
6. Please list about 10 key elements of characteristics that doctors of the Fourth Industrial Revolution should have, and number them from 1 to 10 in order of importance.

| No. | Element | Description of the element | Rank |
| --- | --- | --- | --- |
| Example | Service | The spirit of sacrifice to serve others | 2 |
| 1 |  |  |  |
| 2 |  |  |  |
| 3 |  |  |  |
| 4 |  |  |  |
| 5 |  |  |  |
| 6 |  |  |  |
| 7 |  |  |  |
| 8 |  |  |  |
| 9 |  |  |  |
| 10 |  |  |  |
